# Differential inflammatory responses to acute exercise and *ex vivo* immune challenge in young and master athletes

**DOI:** 10.3389/fimmu.2025.1601405

**Published:** 2025-07-31

**Authors:** Luciele Guerra Minuzzi, Alexandre Abilio De Souza Teixeira, Caique Figueiredo, Gilson Dorneles, Anna Cláudia Castelo Branco, Bruna Spolador de Alencar Silva, Pedro L. Valenzuela, Alessandra Peres, Alejandro Lucia, Maria Notomi Sato, José Cesar Rosa Neto, Karsten Krüger, Fabio Santos Lira

**Affiliations:** ^1^ Exercise and Immunometabolism Research Group, Postgraduation Program in Movement Sciences, Department of Physical Education, Universidade Estadual Paulista “Julio de Mesquita Filho”, Sao Paulo, Brazil; ^2^ Department of Exercise Physiology and Sports Therapy, Institute of Sport Science, Justus-Liebig-University Giessen, Giessen, Germany; ^3^ CIPER, Faculty of Sport Sciences and Physical Education, University of Coimbra, Coimbra, Portugal; ^4^ Immunometabolism Research Group, Institute of Biomedical Science, University of São Paulo, São Paulo, Brazil; ^5^ Escritório de Projetos, Hospital Moinhos de Vento, Porto Alegre, Brazil; ^6^ Laboratory of Cellular and Molecular Immunology, Federal University of Health Sciences of Porto Alegre (UFCSPA), Porto Alegre, RS, Brazil; ^7^ Laboratory of Medical Investigation-56, Department of Dermatology, Faculty of Medicine, University of São Paulo, São Paulo, Brazil; ^8^ GENUD Toledo Research Group, Centro de Investigación Biomédica en Red Fragilidad y Envejecimiento Saludable (CIBERFES), Instituto de Salud Carlos III, Madrid, Spain; ^9^ Grupo Mixto de Fragilidad y Envejecimiento Exitoso UCLM-SESCAM (IDISCAM), Faculty of Sport Sciences, University of Castilla-La Mancha, Toledo, Spain; ^10^ Physical Activity and Health Research Group (PaHerg), Research Institute of Hospital 12 de Octubre (imas12), Madrid, Spain; ^11^ Department of Sports Sciences, Faculty of Medicine, Health and Sports, Universidad Europea de Madrid, Madrid, Spain

**Keywords:** inflammation, athletes, moderate exercise, natural killer cells, monocytes

## Abstract

**Background:**

Lifelong exercise is associated with beneficial immune adaptations, but the extent to which these adaptations manifest during an acute inflammatory challenge remains unclear. Therefore, we aimed to compare the inflammatory responses to ex vivo whole blood and peripheral blood mononuclear cells [PBMCs] cultures from young and master athletes, before and after a single bout of moderate-intensity exercise.

**Methods:**

Young (n=7; 22 ± 4 years) and master (n=12; 52 ± 9 years) female and male athletes with similar performance levels performed a 30-minute bout of moderate-intensity exercise. Blood samples were collected before and post-exercise to assess cytokine production in whole blood and PBMCs after stimulation with lipopolysaccharide [LPS] and a cocktail with phorbol 12-myristate 13-acetate [PMA] plus ionomycin.

**Results:**

In whole blood, LPS induced higher interleukin [IL]-6 release in both groups, with a greater increase in young athletes at post-exercise (p=0.014). Tumor necrosis factor [TNF]-α levels increased only in young athletes (p<0.0001). In PBMCs, master athletes showed lower LPS-induced TNF-α release, increasing only post-exercise (p<0.034), whereas young athletes responded at both baseline (p<0.001) and post-exercise (p=0.003). Under PMA/ionomycin stimulation, TNF-α (p<0.0001) and interferon (IFN)-γ (p=0.007) release increased only in young athletes, while IL-6 production decreased in young athletes at baseline (p=0.002) and post-exercise (p=0.003).

**Conclusion:**

Young athletes exhibit a stronger cytokine response to ex vivo inflammatory stimuli, while master athletes demonstrate a more controlled and regulated inflammatory profile.

## 1 Introduction

The long-term benefits of regular exercise, particularly concerning immune function and inflammation, are mediated—at least partly—by the cumulative effects of repeated bouts of ‘acute’ exercise (1). Of note, although acute unaccustomed exercise can be considered a pro-inflammatory stimulus, it stimulates the release of circulating components—the so-called *exerkines*—that can exert an anti-inflammatory function (2). A well-known example is the exercise-induced increase in IL-6, which induces the release of other circulating cytokines that have anti-inflammatory properties, such as IL-10 (3), while decreasing the circulating levels of the pro-inflammatory cytokine TNF-α (4), thereby ultimately contributing to re-establishing inflammatory homeostasis (5).

Lifelong athletes with consistent and regular years (≥20 years) of exercise practice (also known as ‘master athletes’) represent the paradigm of youthful immunomodulation (as a result of repeated acute exercise bouts in the long term) as they show, among others, similar (e.g., IL-10) if not higher plasma levels of anti-inflammatory cytokines than much younger adults, together with reduced senescent T cell numbers (6–8). Regular exercise may also improve macrophage function by preventing age-related dysregulation in Toll-like receptors (TLRs, which recognize pathogens and activate immune responses to defend against infections) (9). This implies that regular exercise might increase the threshold for immune activation (i.e., the level of stress or stimulus required to activate the innate immune system) (10), potentially making lifelong exercisers more resilient to stress and less prone to excessive or inappropriate immune responses.

However, there is currently no evidence as to whether lifelong exercise also affects the response to a pro-inflammatory stress stimulus, such as exposure to lipopolysaccharide [LPS], a potent endotoxin and widely recognized activator of the monocyte/macrophage arm of the innate immune system—to stimulate cells ex vivo (11, 12). LPS is a component of the outer membrane of Gram-negative bacteria that activates an innate immune response by interacting with Toll-like receptor 4 (TLR4). This interaction initiates a signaling cascade via nuclear factor kappa B (NF-kB), leading to the production of pro-inflammatory cytokines like TNF-α, IL-18, and macrophage inflammatory protein-1α [MIP-1α] required for a robust immune response against bacterial infections (13–15). The normal response to an LPS challenge is characterized by a transient, robust upregulation of inflammatory activity, that resolves once the threat has passed (16). This temporal restriction of inflammation (i.e., activating and resolving the inflammatory response in a controlled manner) is crucial for maintaining overall health, as prolonged or dysregulated inflammatory responses can lead to a host of chronic diseases and health conditions (17). In addition to LPS stimulation, phorbol ester was used in combination with ionomycin, a calcium ionophore, to broadly activate lymphocytes (18). Phorbol 12-myristate 13-acetate (PMA)/ionomycin through protein kinase C (PKC) activates mononuclear cells, including T cells and B cells, and, to some extent, NK cells by mimicking intracellular signaling events that lead to cytokine production and other activation markers (e.g., IFN-γ, CD69).

The primary aim of this study was, therefore, to determine whether master athletes exhibit an inflammatory response comparable to that of younger trained counterparts, with a particular focus on cytokine production. To address this, we compared the cytokine production to pro-inflammatory triggers by stimulating whole blood and PBMC cultures at baseline and after an acute exercise session. By integrating gene expression profiling and detailed flow cytometric phenotyping, we further explored whether differences in cytokine production could be attributable to transcriptional regulation, alterations in immune cell frequency, phenotype, or both. We hypothesized that while younger athletes would demonstrate a robust cytokine release in response to inflammatory stimuli, master athletes would exhibit a comparatively tightly regulated (i.e., controlled yet sufficient) inflammatory response, possibly reflecting adaptive immunomodulatory mechanisms accumulated over long-term regular training.

## 2 Methods

The study was conducted in accordance with the ethical standards of the Declaration of Helsinki. All participants were explained about the purpose and procedures of the study and gave their written informed consent. The Ethical Institutional Review Board approved the study (approval number: 38701820.0.0000.5402).

**Figure 1:**
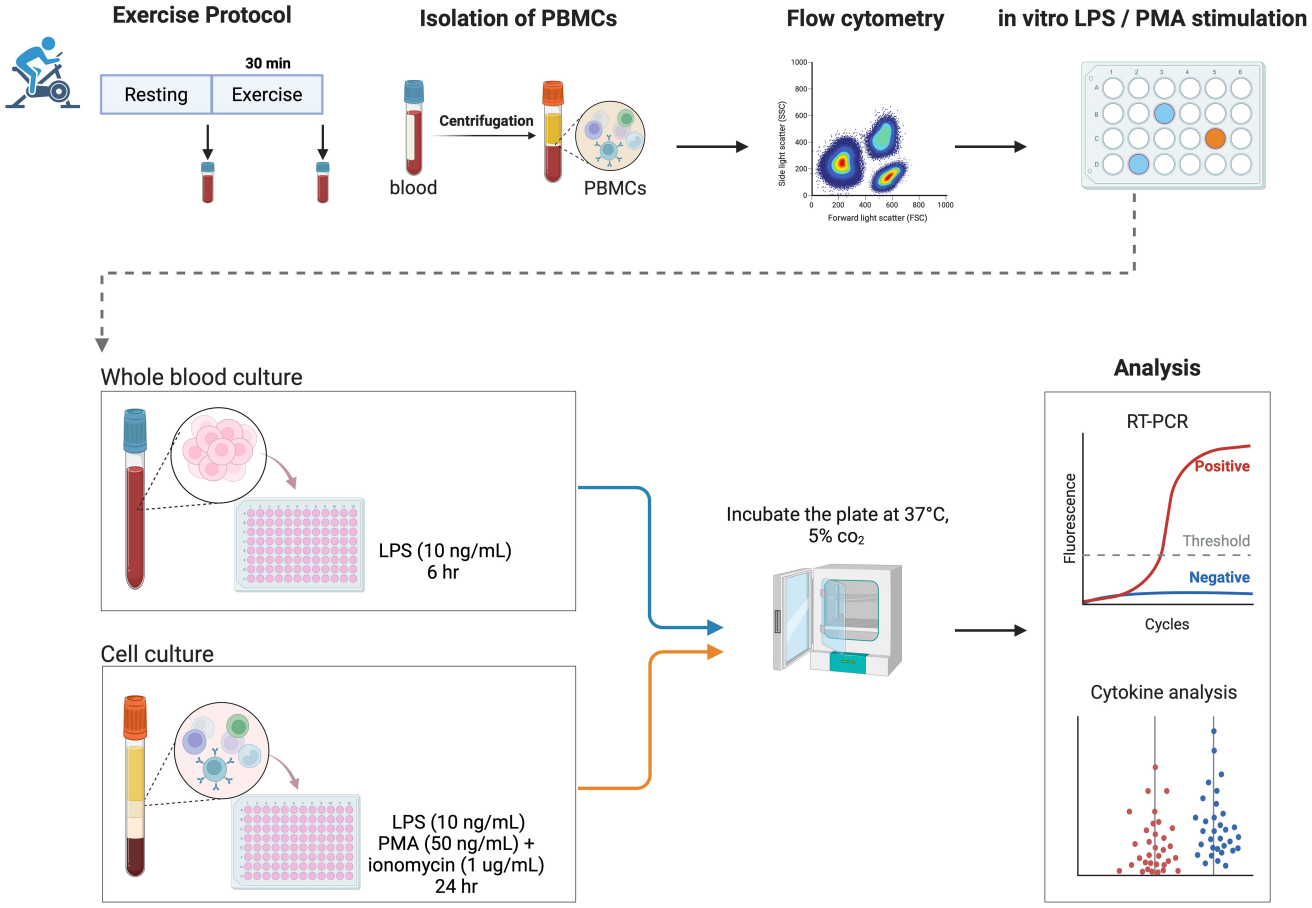
Study design. Overview of the study design, including an acute bout of moderate exercise (30 minutes on the cycle ergometer), blood sampling, isolation of PBMCs, and an *in vitro* inflammatory response protocol in both whole blood and PBMCs. LPS, lipopolysaccharide; PBMCs, peripheral blood mononuclear cells; PMA, phorbol 12-myristate 13-acetate. Created in BioRender. Guerra minuzzi, L. (2025) https://BioRender.com/z58z271.

An overview of the design study is illustrated in **Figure 1**.

### 2.1 Participants

Master athletes (n = 12; 9 men, 3 women; 51.8 ± 9.0 years) included eight cyclists, two track-and-field athletes, and two swimmers with 28.1 ± 13.8 years of continuous training (8.8 ± 4.4 h·wk^-^¹, 11.9 ± 0.3 months·yr^-^¹), whereas young athletes (n = 7; 5 men, 2 women; 22.0 ± 4.1 years) comprised five runners, one cyclist, and one team-sport participant with 8.7 ± 4.1 years of continuous training (9.8 ± 4.9 h·wk^-^¹, 10.8 ± 1.0 months·yr^-^¹). Exclusion criteria included smokers, excessive consumption of alcohol (>2 drinks/day), and any known cardiovascular, musculoskeletal, or neurological condition or regular medication use. The sports experience history of master and young athletes was assessed by self-reporting their athletic life, sports modalities practiced, and the duration dedicated to each category, including the start and end of the year, hours/week, months/year, injuries episodes, and participation in competitions and performances (**Supplementary File 1**). A second questionnaire focused on the development of the other activities, namely type, category, hours/week, and classification of effort intensity, was applied.

Nutritional information was measured at the initial screening using a three-day food diary that consisted of two weekdays and one weekend day to reflect typical intake. Total and relative energy, protein, carbohydrates and lipids intake, and other nutrients (vitamins, minerals, and different types of lipids) were analyzed using NutWinsoftware, version 1.5 (Nutrition Support Program, Federal University of São Paulo, Brazil, 2002) (**Supplementary File 2**).

### 2.2 Exercise protocol

Participants underwent a maximal incremental exercise test on a cycle ergometer (Inbrasport CG-04, Embramed: Porto Alegre, Brazil) linked to an automated breath-by-breath gas analyzer (Quark PFT, Cosmed^®^; Rome, Italy) for peak oxygen uptake (VO_2peak_) and ventilatory threshold (VT) determination. The protocol started with an initial load of 75 W and was increased by 25 W every 3 minutes until volitional exhaustion or until cadence fell below 60 rpm. The VO_2_ peak was determined as the mean VO_2_ consumption in the last 30 seconds of the test. The VT was determined based on the relationship between ventilation (VE) and workload during each stage of the maximal exercise test. Specifically, VE/workload was plotted against workload, and the point at which VE/workload began to rise disproportionately (i.e., a distinct break in the slope) was identified as the VT, in accordance with the method described previously (19). Participants were asked to refrain from vigorous exercise, alcohol consumption, and caffeine for 24 hours before the test. Participants returned to the laboratory at least 24 hours after the aforementioned maximal incremental test to perform a 30-minute exercise bout on the cycle ergometer at a power output slightly above (by 5-15%) the one eliciting the VT in the previous maximal test. The chosen exercise trial is recognized for a selective redeployment of immune cells, notably those subsets with a high differentiation phenotype (20).

### 2.3 Blood measures

All participants arrived at the laboratory following an overnight fast. Upon arrival, baseline venous blood samples were obtained from the forearm vein using Vacutainer tubes (Becton Dickinson, Juiz de Fora, Brazil) containing ethylenediaminetetraacetic acid (EDTA) for subsequent whole blood sampling and isolation of PBMCs, as well as using dry Vacutainer tubes (without anticoagulant agent) for serum separation. Participants then consumed a standardized breakfast that they had brought with them, thereby replicating their typical pre-training meal, and undergoing body composition assessments. Following this period (approximately 1 hour), they completed the acute exercise session. A second blood sample was collected immediately (≤5 minutes for maximal leukocyte mobilization (21)) post-exercise in accordance with the same procedure. Blood samples were centrifuged at 3,000 rpm for 15 minutes at 4°C and the serum supernatant was stored at −80°C until further analysis. Serum levels of IFN-α (i.e., a marker of antiviral response), IL-10, IL-18, MIP-1α, and cytomegalovirus (CMV) were determined by enzyme-linked immunosorbent assay (ELISA) (Duoset R&D System, Minneapolis, MN). All athletes were tested for CMV since studies have shown that CMV influences the immune response (22–24).

### 2.4 Inflammatory response

#### 2.4.1 Whole blood stimulated with LPS

We employed a protocol similar to that described by Barry et al. (25) for the whole blood-stimulated ex vivo assay. Fresh EDTA blood samples were diluted 10-fold with serum-free RPMI media (Sigma Aldrich, MA) supplemented with penicillin (100 U/mL) and streptomycin (0.1 mg/mL), and seeded into a 24-well plate, either in the absence (i.e., ‘non-stimulated’ condition) or adding 10 ng/mL of LPS (*Escherichia coli*, type: 0111: B4; Sigma-Aldrich, St. Louis, MO) (i.e., ‘LPS-stimulated’ condition). Following incubation for 6 hours, the culture supernatants for both non-stimulated and LPS-stimulated conditions were collected and stored at −80°C until further analysis of the inducible IL-6 and TNF-α levels by ELISA (Duoset R&D System, Minneapolis, MN).

#### 2.4.2 Peripheral blood mononuclear cell culture

The EDTA-coated blood samples were added to Histopaque^®^-1077 (Sigma–Aldrich Co. LLC) (1:1) for PBMC isolation and centrifuged at 400×g for 30 min at room temperature. After centrifugation, the PBMCs were washed in phosphate-buffered saline and re-suspended in a supplemented RPMI medium [glutamine (2mM), HEPES (20mM), 10% fetal bovine serum and antibiotics penicillin (100 U/mL) and streptomycin (0.1 mg/mL)] in 12-well plates (Kasvi; PR, Brazil). A total of 1x10^6^ cells were plated either without (non-stimulated) and with (LPS–stimulated) 10 ng/mL of LPS, or with PMA [50 ng/mL] (Sigma, St. Louis, MO) plus ionomycin [1 μg/mL] (Sigma, St. Louis, MO) in a final volume of 1 mL of supplemented RPMI medium. After 24-hour incubation, supernatants of non-stimulated, LPS-stimulated, and ‘PMA/ionomycin-stimulated’ conditions, respectively, were collected and stored at −80°C for subsequent analysis. LPS-stimulated samples were analyzed for IL-6, IL-10, and TNF-α levels, and PMA/ionomycin-stimulated samples were analyzed for IL-6, IFN-γ, and TNF-α levels via ELISA kits (Duoset R&D System, Minneapolis, MN).

### 2.5 Real-time-polymerase chain reaction

Polymerase chain reaction (PCR) was used to amplify and quantify specific genes relevant to inflammation and immune regulation to determine whether exercise status influenced their transcriptional responses following stimulation. Thus, total RNA was extracted from PBMCs using the Brazol reagent (LGC Biotechnology Ltda.; Cotia, Brazil) following the manufacturer’s recommendations and used for real-time polymerase chain reaction (RT-PCR) analyses. The High-Capacity cDNA Reverse Transcription kit (Applied Biosystems, Thermo Fisher Scientific; Foster, CA) was used to perform the reverse transcription to complementary DNA (cDNA), which was stored at -80°C for subsequent analysis of genes by RT-PCR with Power SYBR Green PCR Master Mix (Applied Biosystems). Primer sequences are presented in **Supplementary File 3**.

The gene expression was quantified using β-actin as an internal control. We performed relative quantification (using the 2-ΔΔCT formula) of the expression levels of *ADRB1* (adrenergic receptor β1), *CD62L* (L-selectin), *CCR7* (C-C chemokine receptor type 7), *FAS* (Fas cell surface death receptor), *ICOS* (inducible T-cell co-stimulator), *PDCD1* (programmed cell death protein 1) and TNF-α genes.

### 2.6 Flow cytometry

Flow cytometry allowed us to identify and quantify specific immune cell subsets (such as monocyte and NK cell populations) and to assess the expression of surface markers (e.g., CD69), thus providing insight into how the composition or activation state of immune cells may differ between age groups and across exercise status. PBMCs were isolated using the above-mentioned protocol and then frozen at −80°C in a solution containing 90% fetal bovine serum and 10% dimethyl sulfoxide (DMSO) until analysis. Cells were thawed by diluting them in pre-warmed RPMI 1640 medium containing 5% FBS and spun at 1500 rpm for 5 min. After centrifugation, the cells were re-suspended, and their viability (>98%) was confirmed using trypan-blue staining (Gibco, Grand Island; New York, NJ).

Subsequently, 2 × 10^5^ PBMCs were stained with monoclonal antibodies conjugated to specific fluorochromes for cell phenotyping: anti-CD14+ FITC, anti-CD16+ Pe, anti-CD69+ Percp-Cy5.5, anti-CD3+ FITC, anti-CD56+ Pe, anti-CD16+ Percp-Cy5.5, anti-CD279+ APC, anti-CXCR3 APC, anti-KLRG1 AlexaFluor 633 (BioLegend, San Diego, CA). Flow cytometry analysis was performed using CellQuest Pro Software (BD Bioscience; Franklin Lakes, NJ) on a FACSCalibur flow cytometer (BD Bioscience), with a minimum of 20,000 events per tube acquired. Lymphocytes were identified based on forward and side scatter profiles. The NK-cells were defined as CD3^−^CD56^+^, and the two major subsets of NK-cells were identified as CD56^+^CD16^−^ and CD56^+^CD16^+^ (CD56^bright^ and CD56^dim^, respectively). The frequencies of activated and terminally differentiated NK cell subsets were defined by early activation markers CD69 and CD57, respectively. NK cells subsets were also identified and characterized according to exhaustion [programmed cell death protein 1 (PD1)], senescence [killer-cell lectin-like receptor G1 (KLRG1)], and migratory [CXC chemokine receptor 3 (CXCR3)] markers. Classical (CD14^+^CD16^−^) and non-classical (CD14^+^CD16^+^) monocyte subsets were identified, and the frequency of CD69^+^ cells was determined in each subset.

### 2.7 Statistical analysis

Data are shown as mean and standard deviation (SD) unless otherwise stated. Baseline comparisons between groups were assessed using the Mann–Whitney U test, given the small sample size and potential non-normality of the data. For the main outcomes, between-group and within-group effects were determined using a mixed-design ANOVA with ‘group’ (young, older) as the between-subject factor and ‘condition’ (baseline, exercise) as the within-subject factor. An additional within-subject factor, ‘stimulus’ [i.e., stimulation (or not) with LPS or stimulation (or not) with PMA plus ionomycin], was included to analyze the differences in response to inflammatory stimuli. To verify the assumptions for ANOVA, we applied Levene’s test to assess homogeneity of variance and Mauchly’s test for sphericity. When sphericity was violated, the Greenhouse–Geisser correction was applied. The Bonferroni *post-hoc* test was only performed within groups when a significant interaction effect was found. Analyses were performed using SPSS (v29, IBM, Armonk, NY), with the significance level set as p ≤ 0.05.

## 3 Results

Master athletes reported 2.4 ± 1.7 competitive events in the past two years, 83.3% engaged in at least one additional sport (4.6 ± 1.6 h·wk^-^¹ of alternative activities), and 50% had experienced an injury in the last two years. Young athletes completed 3.2 ± 1.7 events, 85.7% participated in another sport (7.6 ± 2.6 h·wk^-^¹), and 66.7% reported a recent injury (**Supplementary File 1**). Young and master athletes showed similar values of body mass index (24.1 ± 3.4 and 25.4 ± 2.1 kg/m^2^, respectively, p=0.303), VO_2peak_ (53.0 ± 13.2 and 52.2 ± 10.3 ml/kg/min, p=0.442) and power output at the VT (139 ± 24 and 135 ± 37 watts, p=0.786; **Supplementary File 4**). Regarding dietary intake, young athletes consumed more protein than master athletes (p=0.019). No differences were observed in the consumption of carbohydrates, lipids, cholesterol, fiber, or other vitamins and minerals (**Supplementary File 3**).

### 3.1 Serum cytokine profile

**Figure 2:**
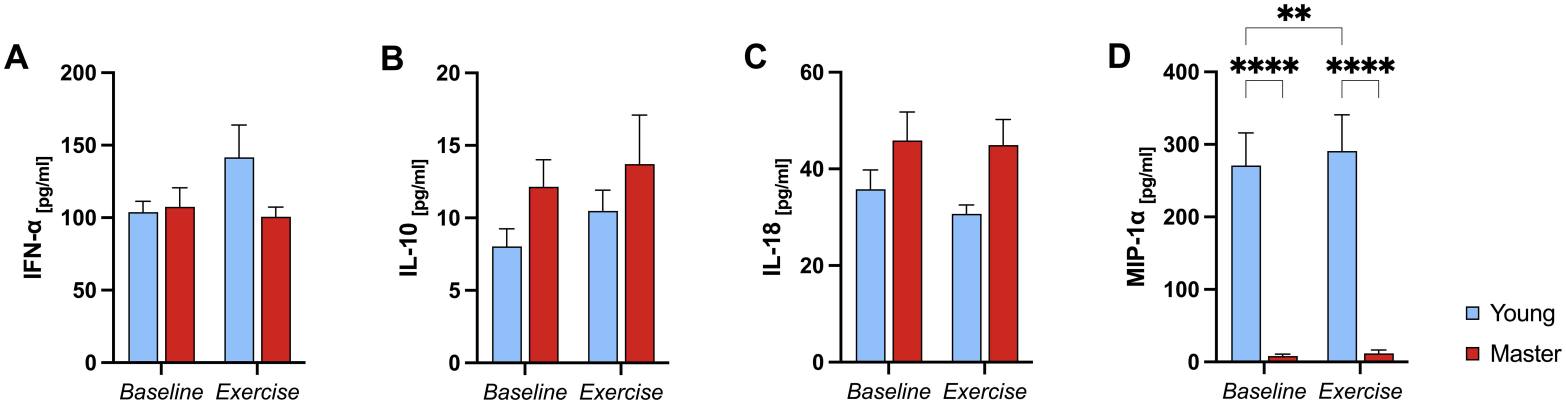
Serum cytokine levels in young and master athletes at baseline and post-exercise. Serum IFN-α **(A)**, IL-10 **(B)**, IL-18 **(C)**, and MIP-1α **(D)** concentrations were measured before (baseline) and immediately after a 30-minute moderate-intensity cycling session (exercise) in young (n = 7) and master (n = 12) athletes. Data are mean ± SEM. **(A–C)**: No significant main or interaction effects. **(D)**: **** p<0.0001, significant Bonferroni post-hoc analysis, two-way ANOVA (group*condition) interaction effect. IFN-α, Interferon-alpha; IL, Interleukin; MIP-1α, Macrophage inflammatory protein-1 alpha.

All participants were CMV seropositive. **Figure 2** presents the serum levels of IFN-α (A), IL-10 (B), IL-18 (C) and MIP-1α (D) measured at baseline and after exercise. A significant group (<0.001), condition (p<0.007), and group*condition interaction (p=0.045) effect was found for MIP-1α (**Figure 2**). Specifically, master athletes had significantly lower MIP-1α levels than young athletes both at baseline (p<0.001) and after exercise (p<0.001). Additionally, in the younger group, MIP-1α levels increased significantly from baseline to post-exercise (p=0.004), whereas no change was observed in the master athletes. In contrast, no significant group, condition, or group*condition interaction effect was observed for IFN-α (p=0.243, p=0.198 and p=0.122, respectively), IL-18 (p=0.148, p=0.150 and p=0.316) or IL-10 (p=0.309, p=0.293 and p=0.823) (**Figure 2**).

In summary, MIP-1α was the only cytokine to show significant differences across groups and exercise conditions—rising in the younger group but remaining consistently lower in master athletes—while IFN-α, IL-18, and IL-10 levels were unchanged regardless of age or exercise.

### 3.2 Frequency of NK cells, classical and non-classical monocytes, and subsets

A significant condition effect was observed for NK cells (p<0.001), but no group (p=0.772) or group*condition interaction effect (p=0.291), indicating that both groups exhibited a similar change from baseline to post-exercise. Similarly, a significant condition effect was observed for PD-1 expression in both NK (p=0.007) and CD56^+^CD16^+^ cells (p=0.020), but no group (p=0.682, p=0.665) or group*condition interaction effect (p=0.731, p=0.258). In contrast, there was no significant group*condition interaction, group or condition effect for CD56^+^CD16^−^ (p=0.393, p=0.558, and p=0.478, respectively) and PD1^+^CD56^+^CD16^−^ (p=0.525, p=0.858, p=0.064) cells. No significant group*condition interaction effects were observed for the expression of CD69, CXCR3, and KLRG1 in NK cells or NK cell subsets (p>0.05) (**Supplementary File 5**). Additionally, there were no significant group, condition, or group*condition interaction effects for the frequency of classical [CD14^+^CD16^−^] (p=0.286, p=0.937, and p=0.255, respectively) or non-classical [CD14^+^CD16^+^] (p=0.253, p=0.937, and p=0.284), monocytes. Likewise, CD69 expression in both classical (p=0.274, p=0.141, and p=0.705) and non-classical (p=0.295, p=0.103, and p=0.762) monocytes did not change with exercise and did not differ between groups (**Supplementary File 5**).

In summary, while total NK cell frequency and PD-1 expression on NK cells and CD56^dim^ subsets showed a significant shift from baseline to post-exercise (indicating a ‘condition’ effect), there were no significant differences between the groups or interactions between group and condition for any cell frequencies or activation markers. Classical and non-classical monocytes, as well as activation markers, remained unchanged across time and between groups.

### 3.3 Whole blood stimulated with LPS

**Figure 3:**
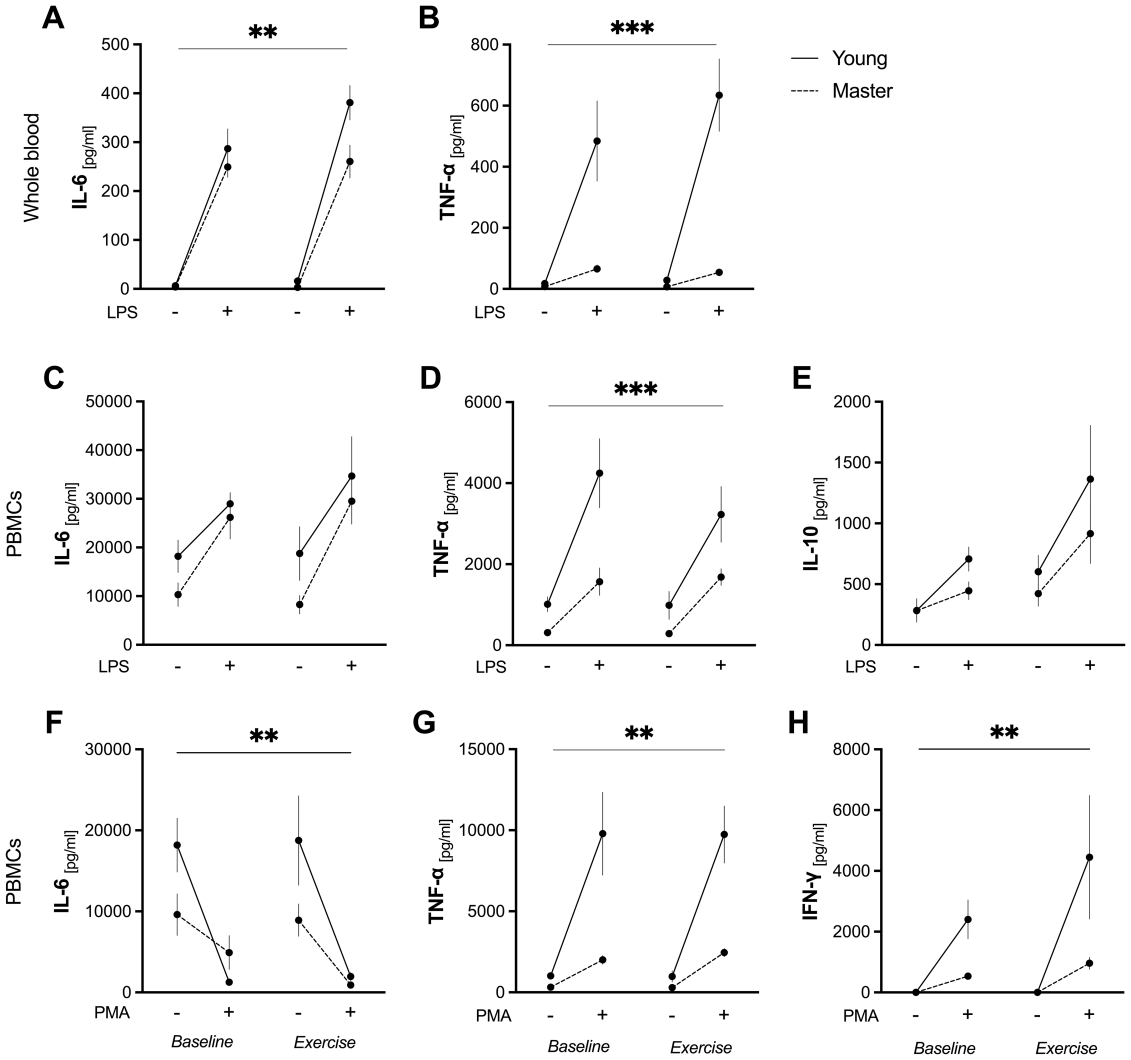
Ex vivo cytokine production in whole blood and PBMCs before and after acute exercise. Whole blood was incubated for 6 h with (+) or without LPS (10 ng/mL) **(A–B)**. PBMCs were stimulated for 24 h with LPS (10 ng/mL) **(C–E)** or PMA (50 ng/mL) + ionomycin (1 μg/mL) **(F–H)**. Data are mean ± SEM for non-stimulated (–) and stimulated (+) conditions in young (n = 7) and master (n = 12) athletes at baseline and immediately after a 30-min moderate cycling bout (“exercise”). **(A)**: LPS-induced IL-6 release in whole blood. ** p<0.05, significant two-way ANOVA (group*condition) interaction effect. **(B, D)**: LPS-induced TNF-α in whole blood **(B)** and in PBMCs **(D)**. *** p<0.05, significant three-way ANOVA (group*condition*stimulus) interaction effect. **(C, E)**: LPS-stimulated IL-6 **(C)** and IL-10 **(E)** release by PBMCs. No significant group, condition, or interaction effects were observed. **(F–H)**: PMA/ionomycin-induced IL-6 **(F)**, TNF-α **(G)**, and IFN-γ **(H)** release by PBMCs. ** p<0.05, significant two-way ANOVA (group*stimulus) interaction effect. IFN-γ, interferon-γ; IL, interleukin; LPS, lipopolysaccharide; PBMCs, peripheral blood mononuclear cells; PMA, phorbol 12-myristate 13-acetate; SEM, standard error of the mean; TNF-α, tumor necrosis factor-α.

A significant group*condition*stimulus (p=0.009), group*condition (p=0.003), and group*stimulus (p<0.0001) interaction effect was observed for TNF-α release in whole blood upon LPS stimulation. Specifically, the younger group showed a robust increase in TNF-α levels in response to LPS at both baseline (p<0.0001) and post-exercise (p<0.0001). In contrast, no significant change was observed in master athletes (p>0.999 for both baseline and exercise). Moreover, master athletes consistently exhibited lower LPS-stimulated TNF-α levels than the younger group a both baseline and exercise conditions (p<0.0001) (**Figure 3**).

No significant group*condition*stimulus interaction effect was observed for IL-6 (p=0.519). However, a significant group*stimulus interaction (p=0.039) effect indicated differential responses between groups when stimulated with LPS. LPS induced a significant increase in IL-6 in both groups at baseline and post-exercise (p<0.0001 for all comparisons). Notably, the younger group showed higher LPS-stimulated IL-6 levels than master athletes in post-exercise (p=0.014) (**Figure 3** **).**

In summary, young adults exhibited a marked increase in LPS-induced TNF-α at both baseline and exercise, while master athletes showed no significant change and maintained consistently lower TNF-α values. Although LPS similarly increased IL-6 in both groups, the younger athletes had higher IL-6 levels post-exercise.

### 3.4 PBMC culture

#### 3.4.1 LPS-stimulated cytokine release

A significant group*stimulus (p<0.034), group (p=0.0001), and stimulus (p<0.0001) effect was observed for TNF-α release by PBMCs upon LPS stimulation, although there was no group*condition*stimulus interaction (p=0.144). In the younger group, LPS significantly increased TNF-α release at baseline (p<0.001) and post-exercise (p=0.003). In contrast, master athletes showed an increase only in the exercise condition (p=0.043). Moreover, LPS-stimulated TNF-α levels were significantly lower in master athletes than in the younger group at baseline (p<0.001), with a trend toward significance post-exercise (p=0.051) (**Figure 3**). These findings suggest that although LPS stimulates TNF release in both groups, young athletes have a more pronounced response, particularly at baseline.

No significant group*condition*stimulus interaction effect was found for LPS-stimulated IL-6 release by PBMCs (p=0.976), and no group*stimulus, group*condition, or condition*stimulus interaction effects were observed (p=0.484, p=0.612, p=0.288, respectively). However, LPS significantly increased IL-6 release in both groups compared to unstimulated conditions (stimulus effect, p=0.0001), and overall IL-6 levels were similar in young compared to the master athletes (group effect, p=0.083). Meanwhile, there were no notable differences when comparing baseline to exercise conditions (condition effect, p=0.444). These data suggest that, while LPS robustly induced IL-6 production, the magnitude of this response differed between the two groups yet remained unaffected by acute exercise. Similarly, there was no significant group*condition*stimulus interaction effect for IL-10 release (p=0.984), and no group*stimulus, group*condition, or condition*stimulus interaction effect (p=0.366, p=0.436, p=0.158) were found. Nevertheless, LPS stimulation significantly increased IL-10 production in both groups (stimulus effect, p=0.013), and IL-10 levels also differed between baseline and post-exercise across all participants (condition effect, p=0.002). However, the pattern of IL-10 release did not differ by group (p=0.231), indicating similar responses to both LPS and exercise (**Figure 3**).

#### 3.4.2 PMA/ionomycin-stimulated cytokine release

No significant group*condition*stimulus interaction effect was found for TNF-α (p=0.724). However, a significant group*stimulus interaction (p<0.0001), group (p<0.0001), and stimulus (p<0.0001) effect were observed. PMA/ionomycin markedly increased TNF-a release in PBMCs from the younger group (p<0.0001), resulting in higher levels than those of master athletes at both baseline and post-exercise (p<0.0001). A similar pattern emerged for IL-6, with no group*condition*stimulus interaction (p=0.506), but a significant group*stimulus (p=0.020) and stimulus (p<0.0001) effect. There was no group (p=0.075) effect. In the younger group, IL-6 production decreased with PMA/ionomycin at both baseline (p=0.0008) and post-exercise (p=0.0009). Conversely, master athletes showed no significant change in IL-6 upon PMA/ionomycin stimulation (p>0.999 for baseline; p=0.134 for exercise conditions; **Figure 3**).

PMA/ionomycin-stimulated IFN-γ release showed no significant group*condition*stimulus interaction effects (p=0.212). However, significant group*stimulus (p=0.007), stimulus (p<0.001), and group (p=0.007) effects were observed. PMA/ionomycin significantly increased IFN-γ release only in young athletes (p<0.001), resulting in higher levels compared to master athletes in post-exercise (p=0.001). No group*condition (p=0.221) or condition*stimulus (p=0.067) interaction effects were found (**Figure 3**).

In summary, while LPS stimulation induced increased TNF-α, IL-6, and IL-10 production by PBMCs in both groups to varying degrees, the younger group generally demonstrated a more robust cytokine response. Under PMA/ionomycin stimulation, the younger group again produced higher TNF-α and IFN-γ than master athletes.

### 3.5 Gene expression

A significant group*condition*stimulus interaction effect (p=0.038) was observed for *CD62L* gene expression in PBMCs following LPS stimulation. Specifically, the young group exhibited higher LPS-stimulated *CD62L* mRNA levels at baseline compared to master athletes (p=0.015) and their own post-exercise levels (p=0.049). By contrast, master athletes did not show a significant change in CD62L expression from baseline to post-exercise.

**Figure 4:**
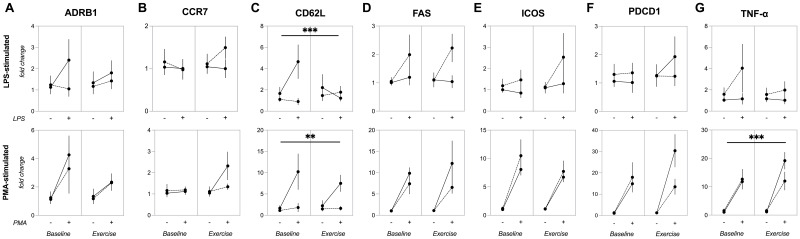
Gene expression of inflammatory and regulatory markers in PBMCs before and after acute exercise. Expression of **(A)** ADRB1, **(B)** CCR7, **(C)** CD62L, **(D)** FAS, **(E)** ICOS, **(F)** PDCD1, **(G)** TNF-α genes in PBMCs stimulated by 24-hour incubation with LPS (upper panels) or PMA plus ionomycin (lower panels). Data are mean ± SEM for non-stimulated (–) or stimulated (+) conditions in young (n=7) and master (n=12) athletes before (baseline) and immediately after a 30-minute moderate cycling session (exercise). **(A, B, D–F)**: No significant main or interaction effects. **(C)** (CD62L): LPS-stimulated CD62L gene expression in PBMCs (upper). *** p<0.005, significant three-way ANOVA (group*condition*stimulus) interaction effect. PMA-stimulated CD62L gene expression in PBMCs (lower). ** p<0.05, significant two-way ANOVA (group*condition) interaction effect. **(G)** (TNF-α): PMA-stimulated TNF-α gene expression in PBMCs. *** p< 0.005, significant three-way ANOVA (group*condition*stimulus) interaction effect. ADRβ1, adrenergic receptor beta-1; CCR7, C-C chemokine receptor type 7; CD62L, CD62L (L-selectin); FAS, cell surface death receptor; ICOS, inducible T-cell costimulatory; LPS, lipopolysaccharide; PBMCs, peripheral blood mononuclear cells; PDCD1, programmed cell death protein 1; PMA, phorbol 12-myristate 13-acetate; SEM, standard error of the mean; TNF-α, tumor necrosis factor-alpha.

No significant group*condition*stimulus interaction was found for PMA/ionomycin-stimulated CD62L expression (p=0.411). However, there were significant group*stimulus (p=0.035), group (p=0.013) and stimulus (p=0.019) effects. In the younger group, *CD62L* gene expression increased at baseline following PMA/ionomycin stimulation (p=0.013), resulting in higher values than those of master athletes (p=0.010). No significant group*condition (p=0.487), condition*stimulus (p=0.258), or condition (p=0.540) effects were observed, suggesting that acute exercise did not influence PMA/ionomycin-induced *CD62L* expression in PBMCs (**Figure 4**).

A significant group*condition*stimulus interaction (p=0.035) effect was found for *TNF-α* gene expression in PBMCs stimulated with PMA/ionomycin, along with a group*condition interaction (p=0.030) and stimulus (p<0.0001) effect. Under PMA/ionomycin stimulation, *TNF-α* gene expression increased for both young and master athletes at baseline (p=0.018 and p=0.053, respectively) and exercise (p<0.0001 and p=0.010). Furthermore, in the younger group, PMA-induced *TNF-α* gene expression was higher in exercise compared to baseline (p=0.015). By contrast, no group*condition*stimulus interaction (p=0.512) was observed for *TNF-α* gene expression when PBMCs were stimulated with LPS (**Figure 4**).

A significant stimulus effect was observed for *ADRB1* (p=0.018), *FAS* (p<0.001), *ICOS* (p<0.0001), and *PDCD1* (p<0.0001) gene expression in PBMCs stimulated with PMA/ionomycin, but not for *CCR7* (p=0.074). Under LPS stimulation, only FAS showed a significant stimulus effect (p=0.043), while *CCR7* (p=0.885), *ADRB1* (0.269), or *PDCD1* (p=0.643) were unaffected. Moreover, there were no significant group*condition*stimulus effects for *ADRB1* (LPS: p=0.303, PMA/ionomycin: p=0.603), *CCR7* (LPS: p=0.186, PMA/ionomycin: p=0.190), *FAS* (LPS: p=0.0.691, PMA/ionomycin: p=0.507), *ICOS* (LPS: p=0.609, PMA/ionomycin: p=0.680), or *PDCD1* (LPS: p=0.348, PMA/ionomycin: p=0.0.068) (**Figure 4**).

In summary, these results indicate that while certain genes (ADRB1, FAS, ICOS, and PDCD1) are responsive to PMA/ionomycin stimulation, and FAS is also responsive to LPS, neither acute exercise nor group differences appear to modulate these effects.

## 4 Discussion

Our findings indicate a distinct inflammatory profile between young and master athletes following acute exercise when evaluated using ex vivo stimulation models, including activation of TLR4 (via LPS) and PKC (via PMA/ionomycin). Overall, young athletes showed a stronger stimulated cytokine response, with higher production of IL-6 and TNF-α in both whole blood and PBMCs cultures. In contrast, master athletes showed lower baseline TNF-α levels across both models but demonstrated an increase in TNF-α production by PBMCs following exercise when stimulated with LPS—an effect not observed at rest—suggesting that the exercise bout modulated their inflammatory responsiveness. These results indicate that master athletes exhibit a more controlled inflammatory response, in contrast to the stronger stimulated cytokine production observed in younger athletes.

Yet, the lower production of TNF-α [a powerful pro-inflammatory agent that is rapidly released after stressors (e.g., LPS) and is one of the main early mediators in inflamed tissues] observed in master athletes could be attributed to a “hypo-responsiveness” state of PBMCs to pro-inflammatory stimuli, such as the so-called ‘endotoxin tolerance’ (15, 26, 27). Endotoxin tolerance, typically characterized by reduced production of pro-inflammatory cytokines upon repeated LPS encounters, can protect against excessive inflammation but may also increase the susceptibility to secondary infections due to immunosuppression. Aging is known to impact immune function, including alterations in cell-type-specific signaling pathways and expression of receptors (14, 28, 29)​​​, thereby impairing endotoxin tolerance (15). Although older adults can exhibit insufficient or exaggerated inflammatory responses in different contexts, our master athletes—who maintain decades of exercise training—seem to exhibit a more regulated inflammatory response rather than an outright deficiency, as suggested by their capacity to upregulate TNF-α post-exercise.

While specific studies on the effects of physical exercise on endotoxin tolerance in aging are limited, these findings align with the literature indicating that regular exercise training can modulate TLR expression and dampen excessive TNF-α release following LPS stimulation (30–32). A 3-week running intervention of moderate intensity prevented excessive immune system activation under LPS-induced inflammatory conditions in mice (32). The training status also exhibits effects on inflammatory response. Antunes et al. found that the anti-inflammatory response is linked with higher physical fitness and may be explained by an efficient cellular framework for the PPAR-γ responses in monocytes (33). In this context, Olesen et al. observed that exposure to LPS-induced inflammation in humans led to a significant suppression of LPS-induced TNF-α levels in trained individuals compared to untrained individuals (31). In the context of acute exhaustive exercise, a decrease in plasma TNF-α levels in mice in response to LPS was attributed to the suppressive impact of this type of effort on the inflammatory response (34, 35). Here, despite a similar proportion of circulating monocytes at baseline and after the acute exercise session, master athletes showed an increased release of TNF-α by PBMCs when stimulated with LPS only in the exercise condition, suggesting that acute exercise impacts the production of cytokines in response to LPS in master athletes.

MIP-1α (CCL3) levels, which attract monocytes and other immune cells to inflammatory sites, were consistently lower in master athletes at both baseline and exercise conditions. On the other hand, MIP-1α levels increased only in the younger group after acute exercise. Although high MIP-1α levels may help recruit immune cells rapidly to sites of damage or infection, chronically elevated levels could contribute to tissue damage or prolonged inflammation. Previous work suggests that regular exercise may reprogram macrophages toward an anti-inflammatory phenotype (e.g. M2-like, typically involved in the removal of tissue debris, tissue remodeling and repair and responsible for large production of IL-10-associated genes), potentially decreasing LPS-induced NF-κB activation and reducing pro-inflammatory gene expression (36). Furthermore, monocytes from individuals with higher physical fitness levels showed a more efficient cellular framework than those with lower physical fitness levels (33). This shift in master athletes might explain the relatively restrained TNF-α and MIP-1α responses in the present study, without completely compromising the ability to mount an inflammatory response when needed (37).

Corroborating with this scenario, we also observed that LPS stimulation increased IL-6 in whole blood from both age groups, but this response was larger in young athletes at post-exercise. Muscle-derived IL-6 is known to shift macrophages toward an anti-inflammatory phenotype (38–40). Hence, the attenuated rise in master athletes could reflect an adaptative, more balanced inflammatory milieu honed over years of regular training (6, 9, 41–46). Similarly, under PMA/ionomycin stimulation, young athletes showed greater IFN-γ and TNF-α production in PBMCs, whereas master athletes showed minimal changes. While such a reduced cytokine release might raise concerns about “exhaustion” (47), neither IL-10 nor PD-1 (PDCD1 gene) was significantly elevated in master athletes. Immune exhaustion is characterized by reduced levels of effector cytokines (such as IFN-γ and TNF-α) and an increase in inhibitory signals [such as IL-10 and PD-1—an inhibitory receptor that can inhibit cell activity upon engagement with its ligands PD-L1 or PD-L2, leading to immune exhaustion (48). We found that IL-10 production by PBMCs, as well as the gene expression of TNF-α and *PDCD1* (gene coding PD-1) were similar between groups. Furthermore, NK-cell markers of senescence or terminal differentiation (such as KLRG1 and PD-1) did not differ between groups, thereby suggesting that the attenuated inflammatory response in master athletes need not imply a pathologic immune exhaustion. Taken together, these findings suggest a comparable capacity to counteract inflammation across ages, which is crucial to prevent ‘excessive’ immune responses (8, 44).

Interestingly, an exercise-specific increase was noted in PD-1 expression on NK cells and CD56^+^CD16^+^ cells, irrespective of age, indicating that acute exercise mobilizes not only effector lymphocytes but also those with a higher cytotoxic profile. As reviewed by Simpson (49) and supported by others (20, 50–52), acute exercise preferentially mobilizes senescent and exhausted T-cells which undergo apoptosis in peripheral tissues during the recovery period (53, 54). This creates an “immunological space” for naïve lymphocytes. Moreover, we observed a stimulus-specific effect on gene expression of FAS—a cell surface-bound receptor that triggers apoptosis (55)—in PBMCs. Whether the exercise-induced increase in PD-1 levels on NK cells causes the apoptosis of these cells during the recovery period (e.g. 1h, 2h) or downregulates NK functionality through enhanced PD-1 signaling needs to be further investigated.

### 4.1 Limitations and future perspectives

Although our strict inclusion criteria have eliminated potential confounding factors (e.g., age-related physical inactivity and elevated fat mass) on the inflammatory response, we acknowledge that the small sample size and unequal distribution of sex and sport disciplines between groups may have limited the generalizability of the findings and introduced variability that could affect assumptions of statistical tests. Future studies with larger and more balanced cohorts are warranted to validate these results. We recognize that the absence of an age-matched untrained control group limits our ability to attribute the observed responses solely to lifelong training without considering the effects of aging in the absence of training. However, from the present results, it cannot be excluded that regular exercise training in master athletes could optimize the ability to respond to stressors (like LPS and PMA stimulation), resulting in lower baseline TNF-α levels compared to young athletes but at the same time increasing TNF-α and IL-6 compared to baseline as part of the inflammatory response. We used a 24-hour stimulation period which may not be optimal for measuring IL-6 levels because it might have resulted in missing differences in the initiation or duration of cytokine secretion (56). This could explain why IL-6 release decreased in PBMCs following stimulation with PMA/ionomycin. Another point which needed be point out is the higher values IL-6, we are acknowledging this limitation and suggesting future experiments to compare PBMC activation markers (e.g., CD69, HLA-DR) and IL-6 mRNA expression post-isolation to determine whether early cell activation contributes to the observed findings.

While individual environmental exposures were not quantified (57, 58), our cohort trained under broadly similar mild-temperature, low-pollution conditions, and all sampling occurred at 22 ± 1°C and 50 ± 5% relative humidity—thus reducing the likelihood that external stimuli (heat, cold, or airborne irritants) systematically influenced our assays. Although our master athletes were free of diagnosed inflammatory diseases and showed no elevations in baseline IFN-, IL-18, or IL-10—and even lower MIP-1α compared to young athletes—subclinical low-grade inflammation cannot be entirely excluded. Future work incorporating other aging-related biomarkers would further delineate the interplay between chronological aging and exercise-induced immune adaptations. Further investigations with larger cohorts and including untrained young and older adults would benefit from the present findings to be able to compare age-related vs. training-related immunological adaptations.

## 5 Conclusion

In summary, our findings indicate that master athletes exhibit a generally lower pro-inflammatory cytokine response to ex vivo stimulation compared to younger athletes, while still maintaining the capacity to upregulate certain mediators following exercise (e.g., maintaining IL-6 release and increasing LPS-induced TNF-α post-exercise). These results suggest a pattern of more controlled inflammatory response in master athletes, balancing the need to mount an appropriate immune response against the risk of excessive inflammation. Additionally, phenotypic markers of natural killer cell differentiation in master athletes were comparable to those of younger trained individuals. Future research with larger cohorts and age-matched controls is warranted to clarify whether these effects are unique to lifelong exercise rather than a feature of aging.

## Data Availability

The original contributions presented in the study are publicly available. This data can be found here: https://hdl.handle.net/11449/183294.
