# Supplementary material for: Differential inflammatory responses to acute exercise and ex vivo immune challenge in young and master athletes
Source: Front Immunol. 2025 Jul 31;16:1601405. doi: 10.3389/fimmu.2025.1601405 (PMC12350127; doi:10.3389/fimmu.2025.1601405)
Supplement: Supplementary file 1 [file SupplementaryFile1.docx]

**Suppl. file 1.** Sports training experience.

| Variables | Young athletes  (n=7) | Master athletes  (n=12) | P value |
| --- | --- | --- | --- |
| *Sports experience* | | | |
| Years of training | 8.7 (4.1) | 28.1 (13.8) | .004** |
| Hours of training/week | 9.8 (4.9) | 8.8 (4.4) | .657 |
| Number of training months/year | 10.8 (1.0) | 11.9 (0.3) | .004** |
| Number of sports competitions  (in the last 2 years) | 3.2 (1.7) | 2.4 (1.7) | .398 |
| Subjects participating in other sports,  n (%) | 6 (85.7) | 10 (83.3) | .446 |
| Hours of alternative training/week | 7.6 (2.6) | 4.6 (1.6) | .023* |
| History of injury in the last 2 years,  n (%) | 4 (66.7) | 6 (50.0) | .627 |

Note: * p<0.05; ** p<0.01. Data is missing from the total sample size for one athlete in both groups regarding the sport history results.
