# Supplementary material for: Differential inflammatory responses to acute exercise and ex vivo immune challenge in young and master athletes
Source: Front Immunol. 2025 Jul 31;16:1601405. doi: 10.3389/fimmu.2025.1601405 (PMC12350127; doi:10.3389/fimmu.2025.1601405)
Supplement: Supplementary file 2 [file SupplementaryFile2.docx]

**Suppl. file 2.** Dietary intake

| Variables | Young athletes  (N=5) | Master athletes  (N=10) | P value |
| --- | --- | --- | --- |
| Total intake, kcal | 2410.0 (1027.1) | 1865.6 (353.4) | .148 |
| Total intake, kcal/kg | 36.2 (21.9) | 25.5 (6.9) | .171 |
| Protein, g | 123.5 (39.4) | 81.5 (22.0) | .019* |
| Protein, % | 17.7 (9.8) | 15.8 (6.5) | .660 |
| Carbohydrate, g | 212.8 (36.4) | 229.5 (59.7) | .580 |
| Carbohydrate, % | 31.6 (19.6) | 43.8 (14.9) | .202 |
| Lipids, g | 120.1 (96.1) | 69.9 (17.4) | .309 |
| Lipids, % | 31.9 (22.3) | 31.3 (11.3) | .944 |

Note: Data are presented as mean (SD). The mean difference is significant at the level: * p<0.05.
