# Supplementary material for: Differential inflammatory responses to acute exercise and ex vivo immune challenge in young and master athletes
Source: Front Immunol. 2025 Jul 31;16:1601405. doi: 10.3389/fimmu.2025.1601405 (PMC12350127; doi:10.3389/fimmu.2025.1601405)
Supplement: Supplementary file 3 [file SupplementaryFile3.docx]

Suppl. file 3. RT-PCR primer sequences – Homo Sapiens

| **Genes** | **Forward Sequence** | **Reverse Sequence** |
| --- | --- | --- |
| ADRB1 | TTCCTGCCCATCCTCATGCACT | GTAGAAGGAGACTACGGACGAG |
| CCR7 | ACAGCCTTCCTGTGTGGTTTT | TCGTCCGTGACCTCATCTTG |
| CD62L | AGAAGGACCAAGCAAAGCCA | CCAGCAGTCGGTTCCATGAT |
| FAS | CACTGTGACCCTTGCACCAA | AGACAAAGCCACCCCAAGTTA |
| ICOS | TTCTCTTCTGCTTGCGCATT | CCCCTTTCAGCAACTGCAT |
| PDCD1 | GCGGCCAGGATGGTTCTTAG | CACGAAGCTCTCCGATGTGT |
| TNF-α | ACTTTGGAGTGATCGGCCC | ATTGGCCAGGAGGGCATTG |
| β-ACTIN | CCTCGCCTTTGCCGATCC | CATGCCGGAGCCGTTGTC |

Abbreviations: *ADRB1*, adrenergic receptor β1 gene; *CCR7,* C-C chemokine receptor type 7 gene; *CD62L*, CD62L (L-selectin) gene; *FAS*, cell surface death receptor gene; *ICOS*, inducible T-cell costimulatory gene; *PDCD1*, programmed cell death protein 1 (PD-1) gene; *TNF*, TNF-α, tumour necrosis factor-α gene.
