# Supplementary material for: Differential inflammatory responses to acute exercise and ex vivo immune challenge in young and master athletes
Source: Front Immunol. 2025 Jul 31;16:1601405. doi: 10.3389/fimmu.2025.1601405 (PMC12350127; doi:10.3389/fimmu.2025.1601405)
Supplement: Supplementary file 4 [file SupplementaryFile4.docx]

**Suppl. file 4.** Comparison of cardiorespiratory fitness between young and experienced athletes.

| **Variables** | **Young athletes**  **(N=7)** | **Master athletes**  **(N=12)** | **P value** |
| --- | --- | --- | --- |
| *Maximal oxygen uptake test* | | | |
| Power, Watts | 217.9 (65.7) | 216.7 (48.1) | 0.964 |
| Heart rate, bpm | 186.7 (9.9) | 171.8 (11.2) | 0.006** |
| VE, L/min | 131.8 (24.3) | 148.9 (33.5) | 0.256 |
| V̇O2_peak_, mL/min | 3893.7 (727.5) | 4005.4 (916.5) | 0.787 |
| V̇O2_peak_, mL/kg/min | 53.0 (13.2) | 52.2 (10.0) | 0.879 |
| V̇CO2, mL/min | 3812.6 (655.5) | 3988.4 (837.2) | 0.641 |
| *Parameters of the acute exercise session* | | | |
| Mean V̇O2, mL/min | 2758.3 (707.9) | 3013.3 (700.2) | 0.672 |
| Mean V̇O2, mL/kg/min | 37.4 (8.7) | 39.4 (8.7) | 0.506 |
| 30-min load at VT, watts | 139.2 (24.1) | 134.8 (37.0) | 0.786 |
| 30-min load, % maximal power | 68.8 (10.9) | 68.2 (10.9) | 0.914 |

Note: Data are presented as mean (SD). The difference is significant at the level: ** <0.01. Abbreviations = VE: Ventilation; V̇O2_peak_: peak oxygen uptake; V̇CO2: carbon dioxide production; VT: ventilatory threshold.
