# Supplementary material for: Differential inflammatory responses to acute exercise and ex vivo immune challenge in young and master athletes
Source: Front Immunol. 2025 Jul 31;16:1601405. doi: 10.3389/fimmu.2025.1601405 (PMC12350127; doi:10.3389/fimmu.2025.1601405)
Supplement: Supplementary file 5 [file SupplementaryFile5.docx]

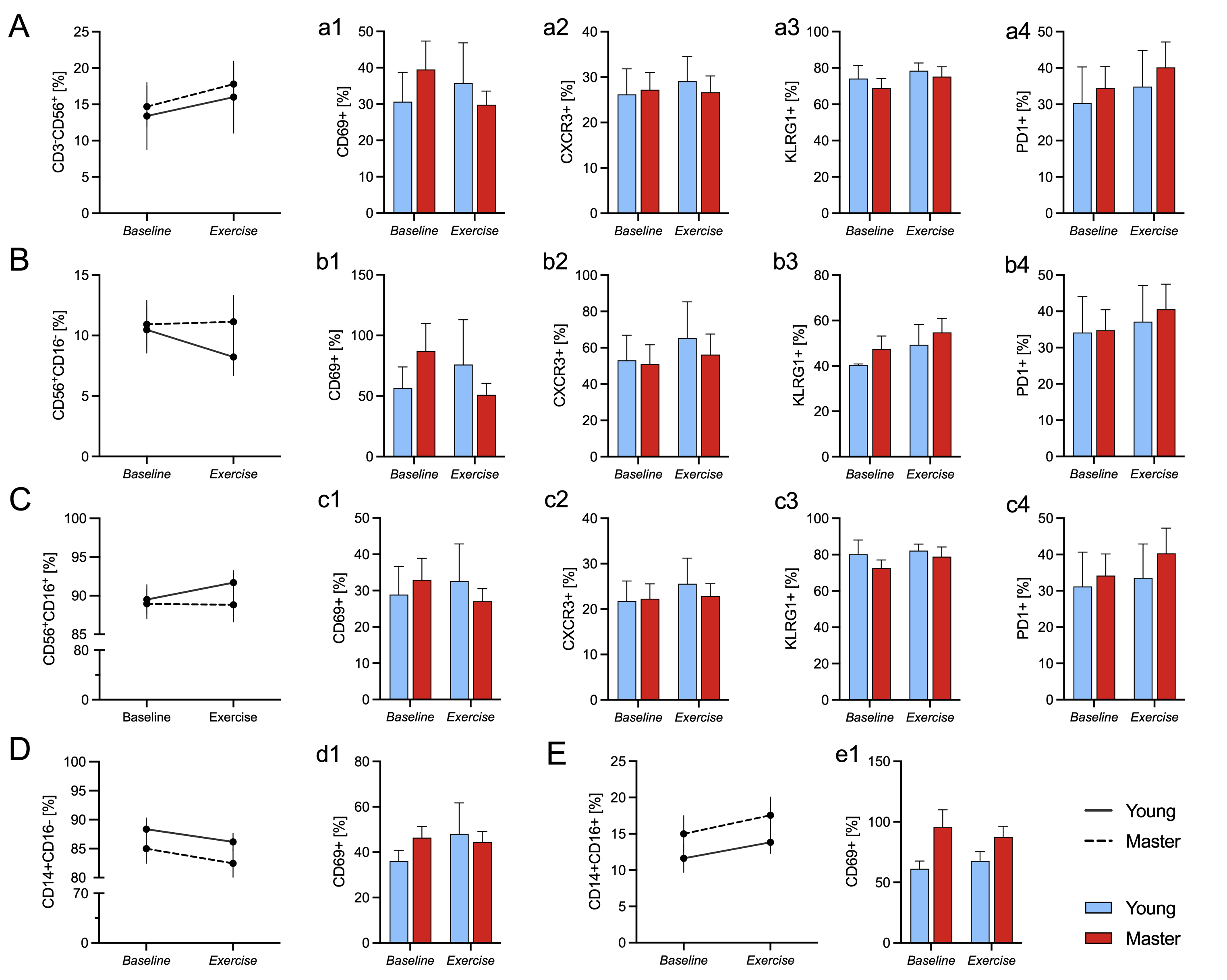


**Suppl file 5. Frequency of NK-cells, classical and non-classical monocytes and subsets**

Values are mean and SEM for non-stimulated (–) or stimulated values for young (n=7) and master groups (n=12) before (baseline) and immediately after 30-min moderate cycling session (exercise).

(A–C) Frequency of total NK cells (CD3⁻CD56⁺; A), CD56+CD16⁻ NK cells (B) and CD56+ CD16⁺ NK cells (C) at baseline and immediately post–exercise. In each panel, the left-hand line graph shows the percentage of the indicated NK-cell population in young (solid line) and master (dashed line) athletes. The adjacent bar graphs (ai–ci) display the surface expression of: CD69 (a1–c1), CXCR3 (a2–c2), KLRG1 (a3–c3) and PD-1 (a4–c4) in young (blue bars) and master (red bars) athletes.

(D) Frequency of classical monocytes (CD14⁺CD16⁻) before and after exercise (line graph) and their CD69 expression (d1, bar graph).

(E) Frequency of nonclassical monocytes (CD14⁺CD16⁺) and corresponding CD69 expression (e1).

Abbreviations: CD, cluster of differentiation; CXCR3, CXC chemokine receptor 3; KLRG1, killer-cell lectin-like receptor G1; PD-1, programmed cell death protein 1.
